# How government health insurance coverage of novel anti-cancer medicines benefited patients in China – a retrospective analysis of hospital clinical data

**DOI:** 10.1186/s12913-021-06840-3

**Published:** 2021-08-21

**Authors:** Yifan Diao, Mengbo Lin, Kai Xu, Ji Huang, Xiongwei Wu, Mingshuang Li, Jing Sun, Hong Li

**Affiliations:** 1 School of Health Policy and Management, Chinese Academy of Medical Sciences & Peking Union Medical College, 5 Dongdansantiao, Dongcheng district, 100730 Beijing, China; 2 Fujian Provincial Hospital, East Street No.134, 35001 Fuzhou, Fujian Province China; 3 Nursing School, Affiliated Clinical Medical Institute of Fujian Medical University, Fujian Provincial Hospital, East Street No.134, 35001 Fuzhou, Fujian Province China

**Keywords:** Novel anti-cancer medicines, Affordability, Benefits package, Interrupted time series study, Binary logistic regression

## Abstract

**Background:**

China started to cover novel medicines for the treatment of major cancers, such as trastuzumab for breast cancer by the government health insurance programs since 2016. Limited data have been published on the use of cancer medications and little is known about how government health insurance coverage of novel anti-cancer medicines benefited patients in the real world. This study aimed to generate evidence to inform the health security authorities to optimize the government health insurance coverage of novel anti-cancer medicines as a more inclusive and equal policy, through which each of the needed patient can get access to the novel anti-cancer medicines regardless of the ability to pay.

**Methods:**

The study targeted one of the government health insurance newly covered novel medicines for breast cancer and the breast cancer patients. The analyses were based on the data collected from one tertiary public hospital in Fujian province of China. We conducted interrupted time series analysis with a segmented regression model and multivariate analyses with a binary logistic regression model to analyze the impact of the government health insurance coverage on medicines utilization and the determinants of patient’s medication choice.

**Results:**

The average proportion of patients who initiated medication with novel medicines increased from 37.4% before the government health insurance coverage to 69.2% afterwards. Such an increase was observed in all patient sub-groups. The monthly proportion of patients who initiated medication with novel medicines increased sharply by 18.3 % (95 %CI,10.4-34.0 %, *p* = 0.01) in September 2017, the afterwards trend continuously increased (95 %CI,1.03–3.60, *p* = 0.02). The critical determinants of patient's medication choice were mostly connected with the patient's health insurance benefits packages.

**Conclusions:**

The government health insurance coverage of novel anti-breast-cancer medicines benefited the patients generally. The utilization of novel medicines such as trastuzumab continuously increased. The insurance coverage benefited well the patients in the high-risk age groups. However, rural patients, patients enrolled in the “resident program”, and patients from low-income residential areas and non-local patients benefited less from this policy. Improving the benefits package of the low-income patients and the “resident program” beneficiary would be of considerable significance for a more inclusive and equal health insurance coverage of novel anti-cancer medicines.

## Background

To improve universal health coverage, it has been increasingly common in emerging countries like Brazil, Mexico and Thailand that the government pools resources and provides financial protection from the catastrophic expenditures for cancer patient, and an increasing number of novel anti-cancer medications have been publicly funded progressively. China achieved universal coverage of basic health insurance coverage through the nationwide health system reforms started in 2009. There are two major separately managed government health insurance programs named the “urban employee program” and the “resident program”. The two  programs cover the urban formal employees with stronger ability to pay, and the non-formally-employed residents in both urban and rural areas with weaker ability to pay respectively. The enrollees of the “urban employee program” and their employers pay fixed proportions of the salary as insurance contributions compulsorily, and entitled to better benefits packages. While the enrollees of the “resident program” receive substantial government subsidies and only pay a very small amount of contribution, but entitled to comparatively weak benefits packages. China started to cover novel anti-cancer medicines by the government health insurance programs in 2016, aimed to improve patient financial access and the quality of cancer treatment. The coverage of novel anti-cancer medicines was built based on the existing benefits packages of the two programs for different population respectively. Although the prices of these newly covered novel medicines for the treatment of major cancers were reduced by more than 50 % [1–4], these medicines might still not be affordable for the patients with poor ability to pay. There have been concerns that the government health insurance coverage of novel anti-cancer medicines might not benefit all the needed patients [5]. However, limited evidence is available about the above concerns, there is a lack of study about the impact of the government health insurance coverage of novel anti-cancer medicines on patients in the real world. Most of the existing evidence estimated the potential change of the medicines expenditure theoretically [6, 7]. Little is known about which population and how much patients benefited from the policy. This study addressed the unmet research needs of the evidence about which part of the targeted cancer patients and how much of them benefited from the public funding of the novel anti-cancer medicines. It presented the changes in the characteristics of the patients who adoptednovel anti-cancer medications before and after the government health insurance coverage. The study also predicted the factors associated with patient’s medication choice based on the real world hospital clinical data, aimed to generate evidence for the health security authorities to optimize the government health insurance coverage of novel anti-cancer medicines as a more inclusive and equal policy, through which each of the needed patient get access to the novel anti-cancer medicines regardless of the ability to pay.

## Methods

### Population and setting

This study targeted trastuzumab which was the only novel anti-breast-cancer medicines covered by the government health insurance and used in the study setting during the observation period. Trastuzumab was recommended for the first-line treatment of human epidermal growth factor receptor 2 positive invasive breast cancer patients in China. As the health insurance programs are managed at the provincial level, different provinces formulate specific policies. This study took Fujian province in Southern China as an example, where the insurance coverage policy of the study medicines was implemented in September 2017 [2, 8]. The study patients were those diagnosed and treated at the provincial medical centre from January 2015 to June 2019 following the Chinese Guidelines for Diagnosis and Treatment of Breast Cancer [9–12]. To reduce the potential selection bias, patients who were diagnosed but not treated at the study hospital, and those who were with contraindications of the study medicines were excluded. Patients with incomplete data were excluded. A total of 357 patients were included in this study.

### Study design

We analyzed the distributions of patients who initiated medication with the study medicines in different demographic, social and economic sub-groups before and after September 2017, which was the time point for the study province started to implement the government health insurance coverage of the study medicines. We compared the proportions of patients who initiated medication with the study medicines before and after periods in each sub-group [9–12].We conducted a longitudinal analysis of the utilization of the study medicines before and after September 2017 which was regarded as the fragmentation of the total study duration. We also performed multivariate analyses to predict the determinants of patient’s medication choice.

### Data source

We extracted the following data from different sources.

(1) Demographic, social and economic information of the study patients were extracted from the database of the front page of the inpatient medical records;

(2) Clinical information was extracted from the electronic medical record database, the medical expense settlement database, and the prescription database;

(3) Disposable income (the amount of money that a household has to spend or save after income taxes have been deducted) of the study patients was obtained from the statistical bulletin of the respective area.

### Measurements

(1) The number and proportion of the study patients who initiated medication with the study medicines before and after September 2017 in different demographic, social and economic characteristics and tumour progression stage sub-groups, were calculated for descriptive analyses;

(2) The monthly proportion of patients who initiated medication with the study medicines before and after September 2017 = monthly number of the study patients who initiated medication with the study medicines / total number of the study patients * 100 %, was used for longitudinal analyses;

(3) Medication choice was defined as “1” for the patients who initiated treatment with the study medicines; “0” for the patients who initiated treatment without the study medicines, was used for the binary logistic regression analyses.

### Statistical analysis

We conducted descriptive statistical analyses of the number of study patients who initiated medication with the study medicines before and after September 2017 in different demographic, social and economic characteristics and tumour progression stage sub-groups. *Chi-square* tests were performed to analyze the differences of the distributions of patients who initiated medication with the study medicines in different demographic, social and economic characteristics and tumour progression stage sub-groups before and after September 2017 respectively. A segmented linear regression model was adopted to analyze the time-series data for the measurement of the level and trend changes of the monthly proportions of patients who initiated medication with the study medicines before and after September 2017 [13, 14]. Finally, we adopted a binary logistic regression model to predict the associated factors of patient’s medication choice before and after September 2017, respectively [15]. The outcome variable was defined as “1” for the patients who initiated medication with the study medicines, and “0” for those who did not adop the study medicines. The patient’s demographic, social, economic characteristics and tumour progression stage were included as covariates in the regression model [9–12, 16]. The number of study patients met the number of sample requirement of the multiple regression analysis with 7 independent variables. The significance level was set as α = 0.05. We used STATA 15 to perform the above analyses.

## Results

As shown in Table 1, among the 357 patients included in the study, 198 patients initiated cancer treatment between January 2015 and August 2017, and 159 patients initiated cancer treatment between September 2017 and June 2019. Among the 198 patients, 74 adopted the study medicines, and 124 did not; and among the 159 patients, 110 adopted the study medicines, and 49 did not. The average proportion of patients who initiated medication with the study medicines increased from 37.4 % before September 2017 to 69.2 % afterwards. The proportions of patients in all sub-groups who initiated medication with the study medicines increased after September 2017. The proportions of patients who initiated medication with the study medicines in each “over 40 years old” group, each “household registration” group, each “level of disposable income” group, each “type of health insurance program” group, each “local and non-local” group, and each “tumour progression stage” group increased after September 2017 (*p* < 0.01).

**Table 1:** The distributions of patients before and after the implementation of the government health insurance coverage in September 2017

Patient characteristics		Before September 2017 (***n***=198)			***P***-value ***(Chi-*** ***square*** test)	After September 2017 (***n***=159)			***P***-value ***(Chi-*** ***square*** test)	Difference between % of patients used the study medicines before and after September 2017 ***p***-value ***(Chi-square*** test)
		No. of patients not used the study medicines (***n***=124)	No. of patients used the study medicines (***n***=74)	The proportion of patients used the study medicines(%)		No. of patients not used the study medicines (***n***=49)	No. of patients used the study medicines (***n***=110)	The proportion of patients used the study medicines (%)		
**Age**	**<40 years old**	6 (4.9)	7 (9.3)	53.8	0.33	4 (0.08)	12 (10.9)	75.0	**0.03**	0.27
	**40-49 years old**	30 (24.4)	21 (29.2)	41.2		8 (16.3)	36 (32.7)	81.8		<0.01
	**50-59 years old**	49 (39.8)	30 (40.0)	38.0		21 (42.8)	42 (38.1)	66.7		<0.01
	**>60 years old**	39 (30.9)	16 (21.3)	29.1		16 (32.6)	20 (18.2)	55.6		<0.01
**Household registration**	**Urban**	46 (37.4)	40 (53.3)	46.5	**0.03**	30 (61.2)	69 (60.9)	69.7	0.96	<0.01
	**Rural**	78 (62.6)	34 (46.7)	31.3		19 (38.7)	41 (39.1)	68.3		<0.01
**Level of disposable income of the patient residential area**	**Low**	47 (39.4)	16 (21.3)	25.8	**0.01**	17 (34.6)	34 (30.9)	66.7	**0.04**	<0.01
	**Middle**	52 (42.3)	33 (45.3)	39.5		18 (36.7)	38 (34.5)	67.9		<0.01
	**High**	25 (20.3)	25 (33.3)	50.0		8 (16.3)	38 (34.5)	82.6		<0.01
**Types of health insurance program**	**Urban employee**	30 (24.4)	30 (40.0)	50.0	**0.02**	20 (40.8)	52 (47.3)	72.2	0.32	<0.01
	**Urban/Rural residents**	76 (61.0)	35 (48.0)	32.4		15 (30.6)	43 (39.1)	74.1		<0.01
	**Non-insured**	18 (14.6)	9 (12.0)	33.3		9 (18.3)	15 (13.6)	62.5		0.04
**Local patients**	**Yes**	76 (61.8)	51 (68.0)	40.2	0.38	24 (49.0)	70 (63.6)	74.5	0.17	<0.01
	**No**	48 (38.2)	23 (32.0)	33.8		25 (51.0)	40 (36.4)	61.5		<0.01
**Tumour progression stage**	**I**	18 (14.6)	14 (18.7)	43.8	0.76	9 (18.3)	23 (21.0)	71.8	**0.04**	0.01
	**II**	68 (54.5)	36 (49.3)	35.6		26 (53.0)	60 (54.5)	69.8		<0.01
	**III**	29 (23.6)	20 (26.7)	40.8		14 (28.6)	20 (18.2)	58.8		0.03
	**IV**	9 (7.3)	4 (5.3)	30.8		0 (0.0)	7 (6.3)	100.0		0.01
		124	74	37.4		49	110	69.2		

Notes: 1. The per capita disposable income of patient residential area was classified into three levels. The low disposable income level was defined as less than USD 2 143, the middle disposable income level was defined as between USD 2 143 and USD 5 000, and the high disposable income level was defined as more than USD 5 000;     2. *Chi-square* test was conducted to analyze the differences of the distributions of the number of patients who initiated medication with the study medicines in different patient groups, and the difference between the proportion of patients who initiated medication with the study medicines in each subgroup before and after September 2017, the significance level was set 0.05

There was no statistically significant difference in the age distribution of the patients who initiated medication with the study medicines before September 2017 (*p =* 0.33); the difference became statistically significant afterwards (*p =* 0.03). The proportion of patients who used the study medicines increased after September 2017 in all age groups. The proportion of patients aged 40–49 years old who initiated medication with the study medicines was the highest, while that for the patients over 60 years old was the lowest. The proportion of urban patients who initiated cancer treatment with trastuzumab was higher than the proportion of rural patients before September 2017 (*p =* 0.03). Such rural-urban statistically significant differences no longer existed after September 2017 (*p =* 0.96). The distribution of patient residential area in different disposable income levels was statistically different before (*p =* 0.01) and after (*p =* 0.04) September 2017. Patient residential ares with higher disposable income level has higher proportion of patients to initiate medication with the study medicines. Before September 2017, there was a statistically significant difference (*p =* 0.02) in the distribution of types of health insurance coverage. The proportion of patients who initiated medication with the study medicines was the highest in the “urban employee program” group. The difference in the distribution of types of health insurance coverage was no longer statistically significant afterwards (*p =* 0.32). There was no statistically significant difference between the proportions of the patients who initiated medication with the study medicines before and after September 2017 in both local and non-local medical groups (*p =* 0.38/0.17). The distribution of patients in different tumour progression stages who initiated medication with the study medicines changed from no statistically significant difference before September 2017 (*p =* 0.76) to statistically different afterwards (*p =* 0.04). The proportion of patients in tumour progression stage IV who initiated medication with the study medicines was the lowest before September 2017 and became the highest afterwards.

**Fig. 1:**
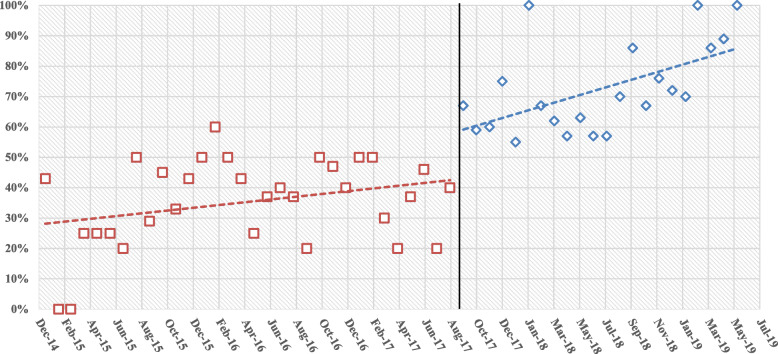
Monthly proportion of patients initiated medication with the study medicines (January 2015-June 2019)

Figure 1; Table 2 showed the level and trend changes in the monthly proportion of patients who initiated medication with the study medicines in 54 months from January 2015 to June 2019. Before September 2017, the trend of the monthly proportion of patients who initiated cancer treatment with trastuzumab was with a slight increase (*p* = 0.08). In September 2017, it suddenly increased by 18.3 % (95 %CI,10.4-34.0 %, *p* = 0.01), and continued to increase after September (95 %CI,1.03–3.60, *p* = 0.02).

**Table. 2:** The segmented regression analysis results of the level and trend changes of the monthly proportion of patients who initiated medication with the study medicines before and after September 2017

Constant 95 %CI(lower, upper)	*p-value*	γ1 195 %CI(lower, upper)	*p-value*	γ2 295 %CI(lower, upper)	*p-value*	γ3 395 %CI(lower, upper)	*p-value*
0.22(0.15,0.31)	< 0.01	0.092(0.04,0.17)	0.08	0.183(0.104,0.34)	0.01	1.48(1.03,3.60)	0.02

**Notes**:

γ1indicates the slope of the trend of the monthly proportion of the patients who initiated medication with the study medicines before September 2017;

γ2 indicates the level change of the monthly proportion of the patients who initiated medication with the study medicines within the month of September 2017;

γ3 indicates the slope of the trend of the monthly proportion of patients who initiated medication with the study medicines after September 2017

As presented in Tables 3, by controlling of the other factors, patients in the “50-59 years old” group were less likely to initiate medication with the study medicines than those in the “<40 years old” group before September 2017 (OR = 0.59, 95 %CI,0.17–0.91, *p* = 0.01), which turned to with no statistically significant difference afterwards (*p* = 0.56). Patients in the “>60 years old” group were less likely to initiate medication with the study medicines than those in the “<40 years old” group before and after September 2017 (before: OR = 0.34, 95 %CI, 0.09–0.87, *p* = 0.01; after: OR = 0.62, 95 %CI, 0.15–0.80, *p* = 0.02). The gaps of the likelihoods between patients in the “<40 years old” group and in the other two older age groups to initiate medication with the study medicines narrowed down afterwards.

**Table 3:** The binary logistic regression analysis results of the associated factors of patient medication choice before and after September 2017

Variables		Before September 2017		After September 2017	
		OR (95%CI: lower, upper)	***p-***value	OR (95%CI: lower, upper)	***p-***value
**Age** **(**Ref: <40 years old)	40-49 years old	0.73 (0.20,2.62)	0.73	1.93 (0.57,5.02)	0.20
	50-59 years old	**0.59 (0.17,0.91)**	**0.01**	1.54 (0.36,6.57)	0.56
	>60 years old	**0.34 (0.09,0.87)**	**0.01**	**0.62 (0.15,0.80)**	**0.02**
**Household registration** **(**Ref: Urban)	Rural	**0.52 (0.29,0.74)**	**<0.01**	0.98 (0.47,1.04)	0.99
**Level of disposable income of patient residential area** **(**Ref: Low)	Middle	**3.47 (1.40,8.59)**	**0.01**	1.06 (0.68,3.83)	0.76
	High	**5.76 (1.48,12.17)**	**0.01**	**1.98 (1.42,17.27)**	**0.02**
**Type of health insurance coverage** **(**Ref: Urban employee)	Urban/rural resident	0.50 (0.35,0.79)	0.03	1.08 (0.29,4.00)	0.91
	Total OOP	**0.47 (0.29,0.68)**	**0.01**	**0.47 (0.14,0.96)**	**0.03**
**Local patients** **(**Ref: Yes)	No	0.84 (0.38,1.85)	0.66	**0.28 (0.10,0.83)**	**0.02**
**Tumor progression stage (**Ref: I)	II	0.820 (0.32,1.77)	0.52	0.89 (0.30,2.58)	0.83
	III	1.012 (0.39,2.64)	0.98	**0.77 (0.17,0.96)**	**0.04**
	IV	**0.751 (0.18,0.86)**	**0.02**	/	/

Note: Bold means statistically significant (*p*<0.05).

Before September 2017, patients registered as rural household were less likely to initiate medication with the study medicines than those registered as urban household (OR = 0.52, 95 %CI, 0.29–0.74, *p* < 0.01). There was no statistically significant difference in the likelihoods between the patients registered as urban and rural households afterwards (*p* = 0.99). Patients from high-income area were more likely to initiate medication with the study medicines than those from the low-income area (before September 2017: OR = 5.76, 95 %CI, 1.48–12.17, *p* = 0.01; afterwards: OR = 1.98, 95 %CI,1.42–17.27, *p* = 0.02). The gap of the likelihoods to initiate medication with the study medicines between patients from high- and low-income residential areas narrowed down afterwards. The “urban and rural resident health insurance program” beneficiary was less likely to initiate medication with the study medicines than the “urban employee health insurance program” beneficiary (OR = 0.50, 95 %CI,0.35–0.79, *p* = 0.03) before September 2017, there was no statistically significant difference afterwards (*p* = 0.91). The non-insured patients were less likely to initiate medication with the study medicines than the “urban employee health insurance program” beneficiary before and after September 2017 (before: OR=, 95 %CI,0.29–0.68, *p* = 0.01; after: OR = 0.47, 95 %CI,0.14–0.96, *p* = 0.03). The likelihood of the non-local patients to initiate medication with the study medicines was not statistically different from that of the local patients before September 2017 (OR = 0.84, 95 %CI,0.38–1.85, *p* = 0.66), which turned higher afterwards (OR = 0.28, 95 %CI,0.096–0.834, *p* = 0.02). The gap in the likelihoods between the local and non-local patients who initiated medication with the study medicines was exacerbated afterwards. Patients in the later tumour progression stage were always less likely to initiate medication with the study medicines than those in the earlier tumour progression stage (before September 2017: OR = 0.75, 95 %CI, 0.18–0.68, *p* = 0.02; afterwards: OR = 0.77, 95 %CI,0.17–0.96, *p* = 0.04).

## Discussion

Patients benefited from the government health insurance coverage of novel anti-cancer medicines generally. The average proportion of patients who initiated medication with the study medicines nearly doubled after the insurance coverage. This was consistent with the findings from the interrupted time series analysis results.

Both univariate and multivariate analyses found that the “40–49 years old” and “50–59 years old” age groups benefited more from the insurance coverage policy than the “<40 years old” age group. Epidemiology literature indicates that the morbidity risk of breast cancer is the highest in the “40–59 years old” age group, decreases slightly in the “60–69 years old” age group, but is still at a high level [17, 18]. This implied that the insurance coverage well benefited the patient age groups at a higher breast cancer incidence risk. To figure out the reasons that the “over 60 years old” age group benefited less than the “under 40 years old” age group, we analyzed the types of insurance entollment of patients in the “over 60 years old” age group and compared with that of the patients in all age groups. We found that in the “over 60 years old” age group, the proportion of patients enrolled in the “resident program” (47 %) was much higher than that in the patients in all age groups (18 %). While the proportion of patients enrolled in the “resident program” who used the study medicines (53 %) in the “over 60 years old” age group was much lower than that of those in the patients in all age groups (74 %). The reasons might be associated with both the type of health insurance program factor and the age factor. As the “over 60 years old” age group got retired, who were more price-sensitive and had lower willingness-to-pay for novel high-priced medicines [19–22].

The study also found that gaps of the proportions and the likelihoods of patients to initiate medication with the study medicines between the urban and rural areas, and among the patients enrolled in different health insurance programs diminished after September 2017. This implied that rural patients and patients enrolled in the “resident program” benefited more from the insurance coverage policy than urban patients and patients enrolled in the “urban employee program”. The multivariable analyses also found that the income level of patient residential area always played a critical role in determining patient’s medication choice. Patients from higher-income level residential area were more prone to initiate medication with the study medicines, which was weakened after the insurance coverage. Such a dominant role of the income level to determine patient’s medication choice was associated with the fact that, from one hand, the higher the income level was, the stronger was the financing ability of the health insurance program [23, 24], and the better was patient’s health insurance benefit package. In 2017, the basic health insurance reimbursement annual cap for inpatient care was ranged from US dollar 8 600 to 20 000 in Fujian province, which was directly linked to the economic development level and the financing ability of a specific area. For the less developed area with weak government financing abilities, the annual health insurance reimbursement cap was set at a lower level [25]. Such a link exists in most areas across the country [26].

On the other hand, patients from higher-income level residential area might have stronger willingness-to-pay and ability to pay, and were more likely to initiate medication with the study medicines. This was especially the case before the insurance coverage [27]. However, the role of the ability to pay to determine the patient’s medication choice might be weakened when the insurance coverage was introduced. The difference of the likelihoods to initiate medication with the study medicines between patients from the high- and low-income residential areas narrowed down after the insurance coverage.

Multivariate analyses found that, compared with the local patients, non-local patients benefited insufficiently from the insurance coverage policy. This is because of more complicated procedures for the non-local patients to get the medical expense reimbursed [27, 28]. Besides, the non-local patients might have to bear a higher financial burden attributable to paying the total expenditure out-of-pocket before getting reimbursed, and a considerable amount of indirect expenses for communication and accommodation, as well as consequent reduction of income [29]. A survey of cancer patients in four hospitals in Beijing found that the indirect medical costs of non-local patients accounted for 19 % of their total medical expenses, and the total expenditure of non-local patients was about 10 % higher than that of the local patients [30]. Qiu et al. investigated the difficulties of the non-local patients in getting the novel medicines reimbursed, and found that the inconsistency of the reimbursement policies in different areas, the low patient awareness of the benefits package, and the complicated reimbursement procedures were the main barriers of the non-local patients to access to novel medicines [31]. A survey about the barriers of access to the study medicines in the United States and several emerging markets showed that doctors in a considerable number of countries reported that insurance coverage, availability of medicines, and cost to the patients were the common barriers [32]. After the novel medicines were included in Mexico’s public insurance system, medicines utilization in remote or less developed areas was much lower than that in the areas which were closer to the capital city [33, 34]..

The critical determinants of patient’s medication choice identified by this study included patient’s household registration, type of health insurance coverage, the disposable income level of the patient residential area, and venue of care. All these were connected with the health insurance benefits packages of patients. Although China has achieved universal coverage of the basic health insurance system, there is yet a national level or even a provincial level pooling of health insurance funds. Insurance funds are operated separately for different population-based programs, and the risk-sharing mechanism across the whole population is yet established [35, 36]. A common strategy of different health insurance schemes in different areas has been that the level of the benefits package is associated with the amount of individual health insurance contribution. There are vast disparities of benefits packages among patients enrolled in different health insurance programs, and among patients enrolled in the same health insurance program but in different areas [36–38]. Low-income patients contribute less and are entitled to the relatively weak benefits packages [39]. Since the insurance coverage of novel anti-cancer medicines has been implemented based on the current basic health insurance system, the “urban and rural resident health insurance program” dominated by rural resident and health insurance programs in less developed areas have weaker fundraising capacities, less contribution, and weaker benefits packages. Weak benefits packages affected the medication choice of those with rural household registration, enrolled in the “resident program”, who came from the low-income residential area, and the non-local patients. Thus resulted that, these patients benefited less from the government health insurance coverage of the novel medicines.

There were several limitations of this study. Firstly, this is a single-center study conducted based on the data extracted from a regional medical centre of Fujian Province, which led to relatively small sample size. The inclusion of more samples from more hospitals may help to further reduce the selection bias to a certain extent. Secondly, as the insurance newly covered novel anti-cancer medicines are all expensive, and the health insurance programs monitored the treatment of the covered indications and the expenditures of the covered medicines carefully, this study assumed that all diagnosis and treatment in the study hospital followed the national guidelines. This study analyzed patient’s medication choice without consideration of doctor’s preference of clinical pathway and its potential impact on the utilization of medicines, cost and patient financial burden. Thirdly, this study was conducted based on the hospital health information which does not include the exact disposable income level of the patients and their families. Further in-depth analyses are needed to help understand and quantify the patient financial burden considering both the real world expenditure and the ability to pay of patient.

## Conclusions

The government health insurance coverage of novel anti-breast-cancer medicines benefited patients generally. Utilization of the study medicines continuously increased. The insurance coverage benefited the patients in the high-risk age groups well. However, rural patients, patients enrolled in the “resident program”, patients from the low-income residential areas and non-local patients benefited less from this policy. The critical determinants of patient’s medication choice were mostly connected with patient’s health insurance benefits package. Improving the benefits packages of the low-income patients and the “resident program” beneficiaries would be of a considerable significance for a more inclusive and equal health insurance coverage of novel anti-cancer medicines. Through which, each of the needed patient can get access to the novel anti-cancer medicines regardless of the ability to pay. Possible policies would be of removing or lowering the deductible and increasing the insurance reimbursement cap, as well as building a safety net for the patients with financial hardship and linking such a safety net with the existing basic health insurance system.

## Data Availability

Data and material will be available upon appropriate request from the corresponding author.

## Abbreviations

CI: Confidence interval
OR: Odds ratio
